# Advancements in Diagnostic Methods and Imaging Technologies in Dentistry: A Literature Review of Emerging Approaches

**DOI:** 10.3390/jcm14041277

**Published:** 2025-02-14

**Authors:** Ana Amélia de Magalhães, Ana Teresa Santos

**Affiliations:** 1 Egas Moniz School of Health & Science, 2829-511 Almada, Portugal; atsantos@egasmoniz.edu.pt; 2 Centro de Estudos Internacionais (CEI), Instituto Universitário de Lisboa (ISCTE-IUL), 1649-026 Lisbon, Portugal

**Keywords:** diagnostic, imaging, technologies, artificial intelligence, cone beam computed tomography, biosensors, dentistry

## Abstract

**Introduction**: Recent advancements in diagnostic imaging technologies have significantly improved the field of dental medicine. This review examines these new imaging techniques and their impact on enhancing accuracy, enabling early detection, and facilitating effective treatment planning in dentistry. **Methods**: A bibliometric and content analysis was conducted on 61 peer-reviewed articles retrieved from the Scopus database, published between 2019 and 2024. The selection criteria focused on studies exploring advances in dental diagnosis through innovative imaging methods and personalized techniques for identifying oral pathologies. The bibliometric approach analyzed publication trends, while content analysis categorized emerging technologies and their clinical applications. **Results**: Our findings indicate a notable shift towards integrating cutting-edge technologies, including Cone Beam Computed Tomography (CBCT), artificial intelligence (AI), and biosensors. These advancements have significantly improved diagnostic accuracy, particularly in complex cases such as periodontal diseases, dental fractures, and oral infections. Studies demonstrate that molecular diagnostics and AI-driven algorithms enhance the personalization of treatment plans, optimizing patient outcomes. **Conclusions**: Emerging diagnostic technologies have the potential to enhance both the quality and efficiency of dental care. However, their implementation is challenged by high costs, the need for specialized training, and disparities in access. Future research should focus on refining AI-driven diagnostic models, addressing regulatory considerations, and expanding the clinical validation of novel imaging tools. As these technologies evolve, they are expected to increase diagnostic specificity, leading to more precise, patient-centered treatment approaches. Ultimately, these advancements offer substantial opportunities to transform dental practice by providing faster, less invasive, and more reliable diagnoses.

## 1. Introduction

The field of dental medicine has undergone remarkable advancements with the introduction of new diagnostic and imaging technologies that are reshaping clinical practice. These innovations enable faster, more accurate, and personalized diagnoses. Notably, these emerging technologies have enhanced our understanding of dental and periodontal anatomical structures. This has facilitated the planning of minimally invasive treatments and improved success rates across various clinical interventions. One significant advancement is the use of Multifunctional Anatomical Prototypes (MAPs), which have been incorporated into surgical planning and dental education. These prototypes offer highly detailed three-dimensional models of dental structures, allowing practitioners and students to simulate complex procedures with greater accuracy. Compared to traditional two-dimensional imaging methods, MAPs provide a more realistic representation of oral anatomy, aiding in both preoperative planning and educational training. Pedrinaci et al. (2024) highlight that this technology fosters greater precision in diagnosing and treating conditions such as dental fractures and developmental anomalies [1].

Another key development in dental medicine is the integration of artificial intelligence (AI) in analyzing dental images, including radiographs and cone-beam computed tomography (CBCT) scans. AI algorithms have demonstrated the ability to identify patterns in bone density images with a level of accuracy that often exceeds human capabilities. This ability significantly aids in the early detection of periodontal diseases and implant planning [2]. The implementation of AI in dental diagnostics enhances efficiency, allowing clinicians to optimize their workflow and improve treatment success rates.

For instance, the study by Gowdar et al. [3] underscores the role of AI in recognizing specific characteristics of bone and dental tissue. This supports the personalized planning of interventions such as bone grafts and orthodontic treatments, which are crucial for achieving optimal patient outcomes. In recent years, molecular diagnostic methods have gained prominence, particularly in the use of biomarkers for detecting infections and managing oral inflammatory conditions. The identification of specific biomarkers allows clinicians to monitor the progression of periodontal diseases and predict treatment responses with greater precision. Agwan et al. [4] emphasize that this approach enhances early intervention capabilities, contributing to more personalized dental care.

The integration of molecular diagnostics with AI has the potential to revolutionize disease management by offering individualized treatment strategies based on a patient’s unique biological characteristics. This is particularly significant for managing chronic periodontal conditions, where tailored treatment approaches can significantly impact long-term outcomes. The rapid detection of infections and inflammation is essential for maintaining oral health. Biosensors have emerged as a promising innovation in this area. These devices enable real-time diagnosis of bacterial infections in the oral cavity, facilitating swift and targeted treatment responses.

The ability to detect pathogens at an early stage is crucial for conditions such as dental abscesses and peri-implantitis, where prompt intervention is necessary to prevent complications. Biosensors represent a new era in precision dentistry, offering clinicians a non-invasive, rapid, and highly accurate diagnostic tool [5]. This review examines the impact of emerging diagnostic technologies and innovative imaging methods in the field of dental medicine. The discussion is structured around three primary themes: advances in dental imaging, molecular and personalized diagnostics, and technologies for diagnosing oral infections. Each section explores the benefits and limitations of these innovations, along with the ethical and economic challenges associated with their implementation in clinical practice.

This research provides meaningful contributions with both scholarly and practical significance for dental medicine. Academically, it serves as a comprehensive roadmap, identifying pivotal studies, influential research trends, and key conceptual frameworks that have shaped the field. Additionally, for healthcare policymakers and administrators, the findings offer insights into optimizing resource allocation, strengthening interdisciplinary collaborations, and advancing oral health strategies. Furthermore, this review addresses critical gaps in the existing literature, such as the clinical validation of emerging technologies, accessibility issues, and the need for standardization in AI-driven diagnostics.

This paper comprises five sections. The second section details the methodology used for data collection and selection, as well as the research framework. The third section highlights key findings related to the evolution of the field and its academic discourse. The final sections provide a critical discussion of the results and conclude with an evaluation of future directions for research and clinical application.

## 2. Materials and Methods

### 2.1. Methodology

Bibliometric analysis is a crucial method for both qualitative and quantitative evaluations of scientific literature [6]. It provides a systematic framework for assessing research outcomes, understanding their implications, and identifying emerging trends within specific topics [7]. This approach was chosen over systematic reviews or meta-analyses due to its ability to map research trends and quantify scientific output. While meta-analyses are effective for synthesizing statistical findings from clinical trials, bibliometric analysis identifies influential studies, research gaps, and emerging trends, offering a broader perspective on the development of diagnostic imaging technologies in dentistry. This method has been particularly recognized in fields such as 5G technologies [8], regional studies [9], and dentistry [10], where it has effectively mapped the contributions of countries, institutions, journals, and individual researchers.

In dentistry, bibliometric analysis has been applied across various specialties, including prosthodontics, endodontics, implantology, orthodontics, pediatric dentistry, and periodontology, providing valuable insights for future advancements [11]. In this study, we employed bibliometric methodologies to conduct a comprehensive review of emerging approaches in dentistry, with the goal of identifying current trends in the field [12].

Our bibliometric approach follows the BIBLIO framework, emphasizing methodological transparency and rigorous quantitative analysis [13]. It integrates guidance on scientific mapping techniques [6] and employs comprehensive bibliometric indicators [14]. This ensures clarity, reproducibility, and inclusivity, allowing for a thorough and responsible exploration of the subject matter.

### 2.2. Data Selection

**Figure 1:**
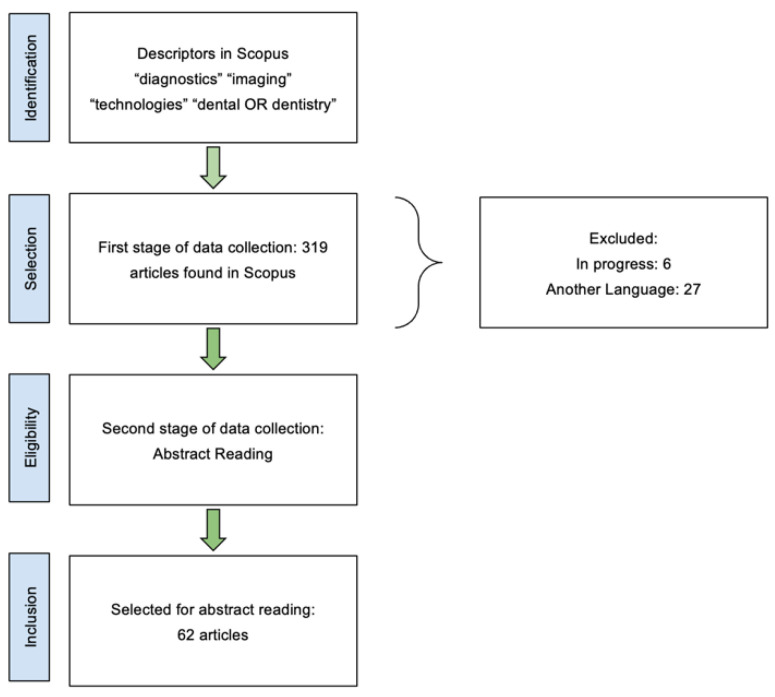
Flowchart of the article selection process in the Scopus database, from initial identification to final inclusion for abstract reading.

For this review, a Boolean search strategy was employed using the keywords “diagnostic AND imaging AND technologies AND dentistry OR dental”. However, this initial query was refined to improve relevance and avoid overly broad results. Additional filters were applied to include only studies that were completed and published between 2019 and 2024, as illustrated in Figure 1.

Further criteria included limiting the results to peer-reviewed journal articles in English, excluding conference proceedings, editorials, and reviews without primary data. This helped to focus on high-quality, research-driven articles that directly contribute to advancements in clinical practice. Scopus was chosen as the primary database due to its established reputation for providing curated, high-quality bibliometric content and its broad multidisciplinary scope, while maintaining rigorous indexing criteria. The decision to use a single database may represent a limitation, as relevant studies indexed in other well-established bibliographic sources might not have been considered.

The decision to exclude other databases was based on prior research indicating that Scopus offers more comprehensive coverage of high-impact scientific literature in dentistry while ensuring rigorous content selection [15]. However, acknowledging possible limitations, future research could incorporate multiple databases to expand the scope of the analysis and address potential selection biases.

A total of 61 peer-reviewed and indexed articles were selected and compiled in December 2024, all concentrating on emerging diagnostic methods and imaging technologies relevant to dental medicine. The inclusion criteria required that the articles be original research studies published in indexed journals, with a clear focus on advancements in diagnostic methodologies in dentistry. Studies that lacked sufficient methodological detail, were not peer-reviewed, or did not contribute directly to the field of diagnostic imaging were excluded.

The analysis was conducted through a comparative review, where each diagnostic technology and method was evaluated for its clinical applicability, diagnostic precision, and relevance to dental medicine. Additionally, a qualitative assessment was performed based on parameters such as technological innovation, ease of clinical implementation, and diagnostic accuracy. This dual approach allowed for a more structured evaluation, ensuring that both technical and clinical aspects were adequately considered. The use of qualitative metrics strengthened the reliability of the findings and enhanced the assessment of emerging diagnostic technologies.

Figure 1 illustrates the search strategy and selection process, detailing the number of initial results, the filtering stages, and the final number of included articles. This step-by-step visualization provides transparency in the selection process and enhances the reproducibility of the study.

## 3. Results

### 3.1. The Development of the Field

#### 3.1.1. The Evolution of the Discipline

**Figure 2:**
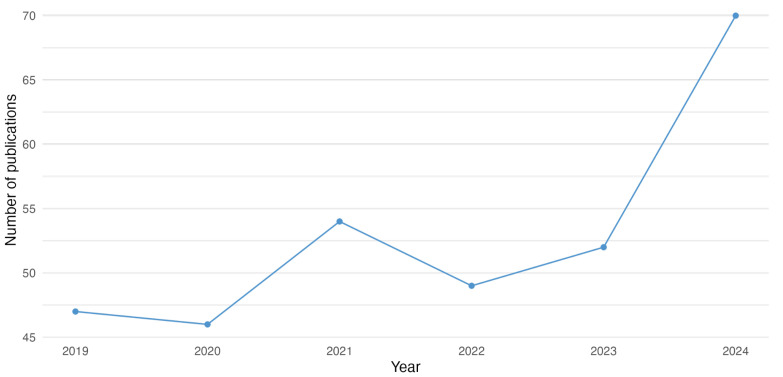
Frequency of papers published per year between 2019 and 2023.

In our review, we analyzed a selection of articles and organized all pertinent publications by year of publication. This method enabled us to monitor the progression of scientific research concerning advancements in diagnostic methods and imaging technologies in dentistry over recent years. Figure 2 depicts the number of publications each year, emphasizing significant trends in research activity.

The publication year of 2024 stands out as the most active, followed closely by 2019 and 2024. These peaks likely indicate periods of robust research activity, fueled by increased funding and advancements in technology. In contrast, the year 2022 experienced a decline in publications, which may reflect quieter phases in the research cycle or changes in priorities and resource allocation.

The abundance of recent articles from 2023 and 2024 is particularly encouraging. It suggests that research is thriving and responsive to the challenges and opportunities of today’s world. These publications not only highlight current progress but also provide insights into the future directions of science.

#### 3.1.2. Publishing Journals

**Figure 3:**
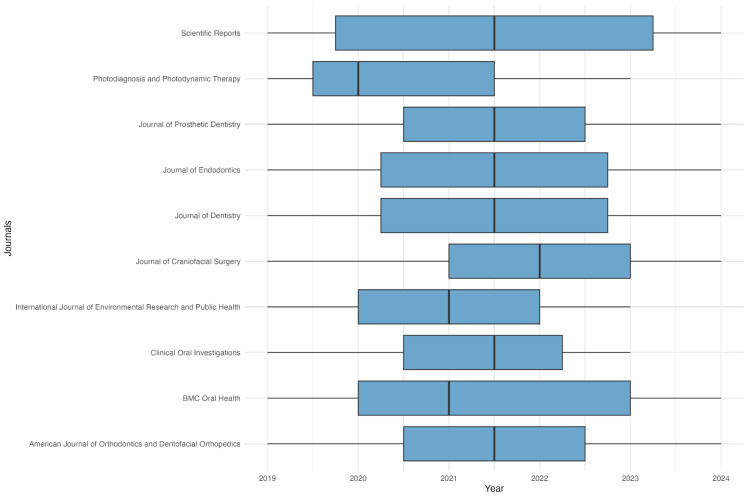
Publication trends of top 10 journals.

From 2019 to 2024, our analysis uncovered 46 journals that have published 61 articles related to the use of AI in dental specialties; precisely a significant portion of these sources have published at least five articles over the past 5 years. The top 10 journals in terms of the number of published articles are listed in Figure 3.

The journals illustrated in the figure above are pivotal to the ongoing advancements in diagnostic technologies within dentistry. Publications such as *Scientific Reports* offer a comprehensive platform for disseminating high-impact research, providing global insights into innovative diagnostic techniques. *Photodiagnosis and Photodynamic Therapy* specifically concentrate on light-based technologies for non-invasive diagnostics, while the *Journal of Prosthetic Dentistry* and the *Journal of Endodontics* investigate the application of advanced imaging in restorative and endodontic treatments, thereby enhancing clinical precision.

Furthermore, journals like the *Journal of Dentistry* and the *Journal of Craniofacial Surgery* underscore the integration of diagnostic technologies into dental and surgical practices, leading to improved treatment planning and better surgical outcomes. The International *Journal of Environmental Research and Public Health* and *BMC Oral Health* highlight the public health implications of these innovations, drawing attention to their influence on global access to dental care. Finally, journals such as *Clinical Oral Investigations* and the *American Journal of Orthodontics and Dentofacial Orthopedics* authenticate the clinical utilization of diagnostic tools, ensuring they adhere to the highest standards for implementation in dental practice. Collectively, these journals propel progress in dental diagnostics, facilitating the refinement and integration of new technologies into clinical workflows.

#### 3.1.3. Geographic Distribution of Authors

**Figure 4:**
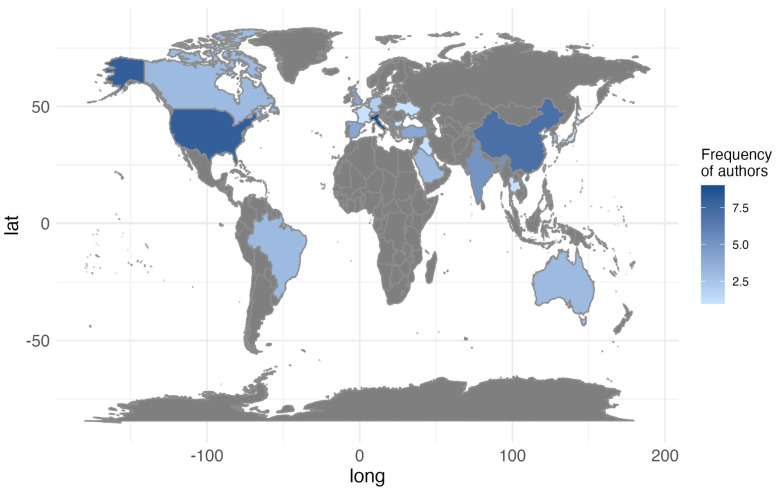
Global distribution of publications (2019–2024).

This section explores the geographic distribution of the articles’ origins. Through an analysis of 61 selected articles and 326 contributing authors, the data offer an overview of global research participation, emphasizing the leading countries in publication output. Figure 4 maps geographic contributions; the aim is to identify regional trends, emerging research hubs, and potential disparities in scientific engagement.

The analysis of the data reveals significant trends in global scientific output regarding advancements in diagnostic methods and imaging technologies in dentistry. The United States, with eight publications, continues to uphold its historical leadership in research. However, emerging countries such as China (seven publications) and India (five publications) are becoming prominent contributors, reflecting their increasing investments in scientific development. Surprisingly, Italy leads with nine publications, indicating a dedicated academic effort or a specialization within this field, which underscores its growing importance in dental research.

Additionally, countries like Brazil, Saudi Arabia, and Turkey are making noteworthy contributions, demonstrating their commitment to enhancing research capabilities. Nations with traditionally limited scientific output, such as Iraq and Macedonia, are also beginning to participate, albeit at a modest level. Conversely, the lack of publications from regions like Africa highlights ongoing disparities in global research participation.

While these findings illustrate a shift towards a more geographically diverse scientific output, significant inequalities persist. The increasing presence of new contributors is promising, yet strengthening international collaboration remains crucial to further expand the reach and impact of scientific research in this field.

### 3.2. The Scientific Discourse

To achieve a comprehensive understanding of the context and methodologies presented in each article, we analyzed the abstracts as concise summaries of the entire paper. This process comprised four essential steps: text segmentation, the removal of numbers and punctuation, conversion to lowercase, elimination of stop words and lemmatization. Each step was designed to ensure data quality and accuracy by minimizing noise (irrelevant or redundant information) and reducing the dimensionality of the data, thus making it more manageable and computationally efficient for analysis.

The initial phase of our process involved text segmentation, also known as tokenization, which entails breaking down the main text into distinct words based on defined word boundaries, such as whitespace. This methodology aligns with the approach outlined by Hinterberger et al. [16]. Subsequently, we converted the entire corpus to lowercase and removed elements such as numbers, punctuation, and running heads to streamline the texts for enhanced analytical precision. In addition, we eliminated what are considered trivial or auxiliary words, commonly referred to as “stop words”, as their presence contributes minimally to our analytical objectives and their removal does not compromise the integrity of the text analysis results [17]. Ultimately, we mapped the 50 most frequent words in each major topic.

The selection of topics addressed in this section was based on their prominence among the themes identified in articles retrieved from the Scopus database, as well as their practical and scientific relevance to dental medicine. The focus on these topics is justified not only by their frequency in the literature but also by their direct impact on clinical practice, enhancing diagnostic efficiency, treatment personalization, and patient outcomes. This approach reflects our effort to identify and discuss areas that embody current and future trends in the field of dentistry.

#### 3.2.1. Diagnosis in Dentistry

**Figure 5:**
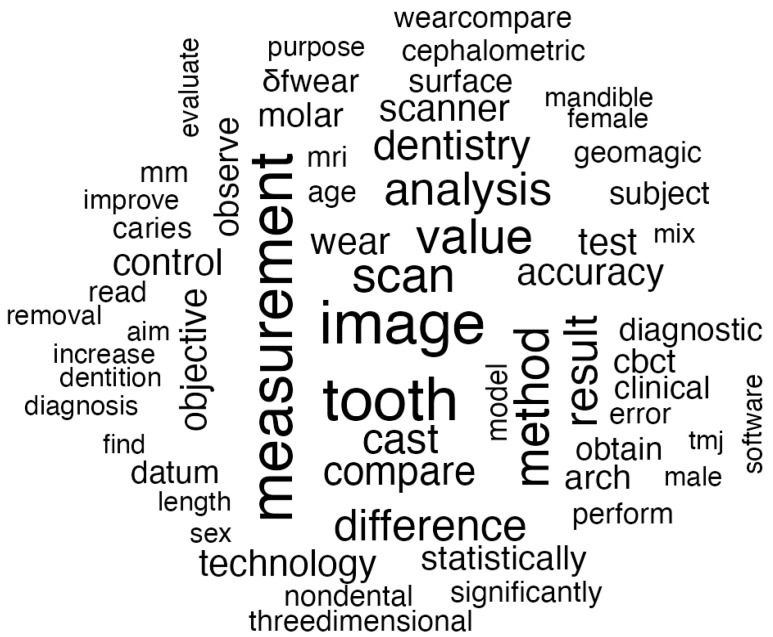
Word cloud visualization of frequently and repeatedly used words about diagnosis.

Diagnosis in dental medicine is one of the most important steps to the efficiency and success of therapeutic intervention. The evolution and rapid development of new technologies in recent years have radically changed the professional approach to early detection within the spectrum of dental pathologies and conditions. Modern equipment offers detailed and accurate diagnoses that lead to personalized treatment with great improvement in both the patient’s experience and the clinical outcome. Figure 5 displays the most common terms found in article abstracts related to diagnosis. Topics that appear more frequently are highlighted with darker, larger font.

The words “image” and “scan” appear as some of the most frequent ones as imaging systems were at the center of the most novel techniques. One of the most significant developments is CBCT. Agwan et al. [4] referred to CBCT as having the potential to provide very accurate three-dimensional imaging capable of yielding an in-depth analysis of bone and tooth structures. The modality is quite handy in showing a large gamut of conditions, starting from dental implant placement to surgical procedures and apical lesions. Within the same context, Chen et al. [5] support this opinion by mentioning that CBCT has increased the standards of diagnostic quality in the case of complicated dental trauma.

In particular, the latest improvements in computational methods have enhanced current diagnostic capabilities to obtain more accurate “measurements” in the scope of dental medicine. Recent studies [4,5] revealed that the developed systems with state-of-the-art computational approaches could diagnose radiographic patterns, almost on par with expert assessments. It therefore not only accelerates the diagnostic process but also reduces the possibility of errors and hence increases the level of confidence for both the professionals and the patients. Additional studies have also documented that these techniques have been particularly successful in delineating benign from malignant lesions [3].

On the other hand, the 3D printing technology has enabled dental practitioners to fabricate precise anatomical models that assist in diagnosis and treatment planning. Multifunctional anatomical prototypes enable a “three-dimensional” understanding of complex cases, which are of particular importance during surgical interventions and in the rehabilitation of highly deformed individuals [1]. In that direction, 3D printing increased diagnostic precision and helped teams to decide in collaboration with each other. [3]

Another highly relevant domain of ongoing attention is represented by the early diagnosis of periodontal diseases and potentially malignant lesions. Recent studies, as represented by Gowdar et al. [3], underlined the increasing role of high-resolution imaging technologies and predictive “models” within the context of the early recognition of pathological patterns. At the same time, it is also expected that wide perspectives will be opened due to the application of biomarkers at the molecular-genetic level. Pedrinaci et al. [1] added that the merging of computational tools with biomarkers has resulted in an extremely effective hybrid approach within the process of monitoring oral cancer.

Recent advances in diagnostic technology have enabled a more personalized approach to dental medicine with high “accuracy”. The study by Arjun et al. [2] investigates the interaction between new diagnostic systems and new biomaterials, thus favoring an optimal integration of diagnosis and treatment for specific pathologies. Along this line, the study states that the combined use of new diagnostic technologies and personalized therapies greatly increases the possibility of clinical success in rehabilitation with complexity [1].

#### 3.2.2. Dental Surgery

**Figure 6:**
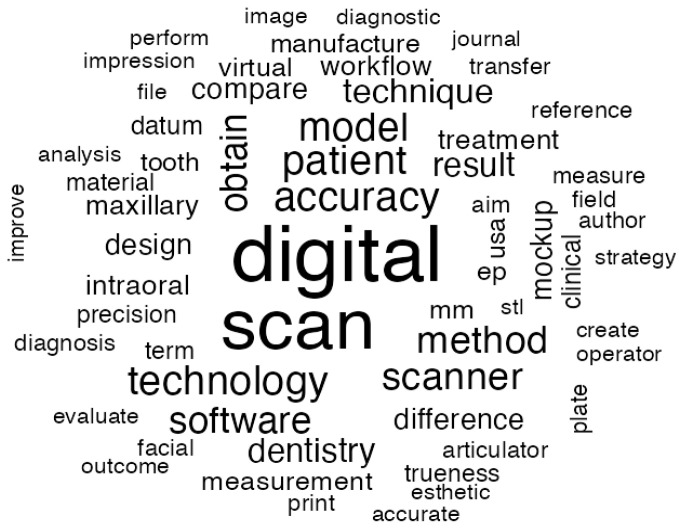
Word cloud visualization of frequently and repeatedly used words about surgery.

Surgical advances in dentistry have drastically changed clinical practices, enabling the achievement of more precise, minimally invasive, and patient-centered approaches. Advanced imaging technologies, robotic systems, and digital tools have enabled a new era in surgical planning and execution. Figure 6 presents the most commonly occurring words found in the abstracts of surgery-related articles. The words displayed in darker and larger fonts represent the most frequently discussed topics.

The “digital” technologies, such as a CBCT “scan”, have played a pivotal role in surgery. Surface texture mapping through CBCT helps in complex surgical planning, especially in cases where a detailed assessment of bone structures is indispensable. [1] This technology ensures that surgeons have a thorough knowledge of the operative field, which reduces risks and improves outcomes. In the same line, Godoy-Santos et al. [18] talk about the use of weight-bearing CBCT, showing its usefulness in the assessment of load-bearing anatomical structures, which is indispensable for the exact placement of implants.

More attention has been given to novel methodologies like haptic robotic systems. Ali et al. [19] details a case report on the use of haptic “technology” in flapless dental implant surgery. This combined “technique” of adding tactile feedback to robotic precision enables surgeons to perform complex procedures with more accuracy and less trauma to patients. These advancements not only maximize the process of surgery but also fast-track recovery times for patients.

Preoperative planning has been further revolutionized by “digital” face “scanning” and morphometric analysis. Amornvit et al. [20] highlight the accuracy of digital face scans in generating three-dimensional models of faces for surgical interventions. These “models” can give an overall view of the patient’s facial anatomy, allowing tailoring of the surgical approaches with both functional and aesthetic outcomes in mind. In a related study, Güzel et al. [21] investigate the application of morphometric analysis in the elucidation of craniofacial structures with insights that enhance both surgical accuracy and predictability.

The advancement of minimally invasive procedures has also been supported by innovations in imaging and “digital” technologies. Surface texture mapping combined with CBCT significantly reduces the need for exploratory surgical interventions, ensuring that interventions are both targeted and effective. This aligns with broader trends in dentistry, where the emphasis is on reducing patient discomfort and keeping recovery times to a minimum while maintaining high standards of care [3].

The array of technological advances together underlines the progressive transformation of surgical methodologies within the field of dentistry. The incorporation of technologies like sophisticated imaging, robotic systems, and “digital” instruments contributes to the continuing refinement of modern dental surgical capability. These innovations epitomize the synthesis of cooperative research efforts and clinical proficiency, guaranteeing that surgical procedures are both efficient and consonant with the highest standards of patient care. The future looks bright for oral surgery as further research refines and innovates these milestone methods.

#### 3.2.3. Dental Imaging Advancements

**Figure 7:**
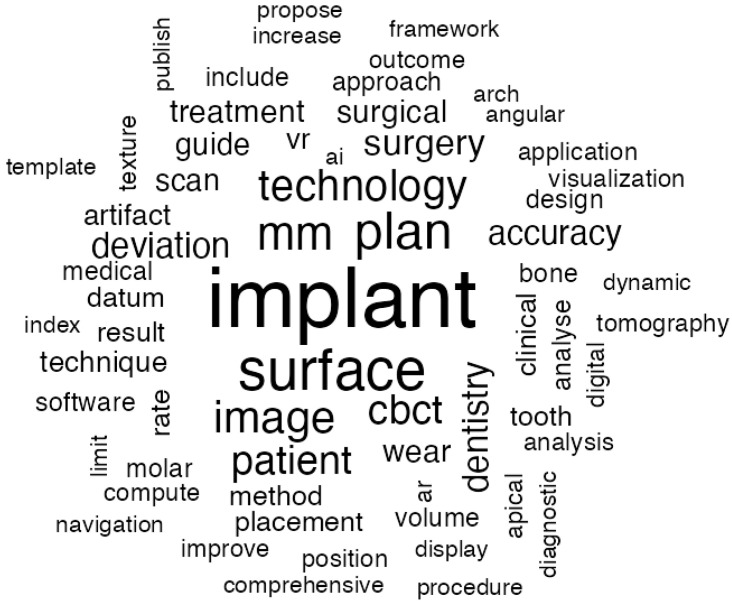
Word cloud visualization of frequently and repeatedly used words about imaging.

Dental imaging has undergone significant development, becoming an important aspect of modern dentistry. Its importance is highly considered in the diagnosis, treatment planning, and follow-up fields due to the development of new technologies and methodologies. Figure 7 illustrates the most frequent words in the abstracts of articles about imaging. Words with darker and larger fonts are more frequent topics.

Digital radiography and three-dimensional imaging “technologies” have revolutionized the way clinicians assess dental structures, mainly for implants. The incorporation of virtual prosthodontic initiatives has shown the huge transformative capabilities of dental imaging. Lavorgna et al. [22] underline the reliability of virtual tools in the preparation and simulation of prosthetic treatments. Such tools would also improve communication between the dental professional and the patient, hence leading to better understanding and more realistic expectations regarding treatment outcomes.

Lo Giudice et al. [23] discuss the use of digital analysis for occlusal changes, periodontal assessment and teeth “surface”. The approach presents an increased level of precision with a reduction in radiation exposure to the patient compared with the traditional approach. Additionally, Puett et al. [24] have introduced synthetic radiography in intraoral imaging, showing increased detail and clarity within the anatomical visualizations, which is important in obtaining a precise diagnosis.

The translation of research findings into clinical applications and therapeutic “plans” has been one of the major focuses of emphasis. Heft et al. [25] emphasize the strides that have been made over the past century in integrating state-of-the-art imaging methodologies into daily dental practice and surgical purposes. Their research shows how systematic efforts at research and development have, over time, successfully translated experimental imaging innovations into their applications in the treatment of patients.

Furthermore, the application of imaging techniques for forensic and anthropological objectives illustrates its adaptability. Meral et al. [26] investigated the estimation of sex utilizing foramen magnum parameters via imaging methods. Their results indicate the capacity of dental imaging to transcend traditional clinical uses, thereby making significant contributions to wider scientific and legal contexts.

AI has also been making inroads in dental “image”, enhancing image interpretation and diagnostic accuracy. While not explicitly mentioned in the cited articles, this trend reflects a broader trajectory where AI systems augment clinicians’ capabilities, offering new possibilities for precision and efficiency.

Recently, the application of intraoral ultrasonography combined with artificial intelligence has shown significant advancements in dental diagnostics, particularly in the analysis of the periodontium and adjacent dental structures. Le et al. [27] developed a compact intraoral ultrasound device featuring a high-frequency (up to 25 MHz) rotational transducer, enabling real-time imaging of intraoral structures, including the dento-periodontium and maxillary palate. The innovation lies in the integration of machine learning algorithms that automate the identification of key structures, such as the alveolar bone and cementoenamel junction, enhancing diagnostic precision and optimizing treatment planning. This technology represents a significant breakthrough in precision dentistry, making diagnostics less invasive and more accessible while improving clinical predictability and the personalization of dental treatments.

#### 3.2.4. Biomaterials

**Figure 8:**
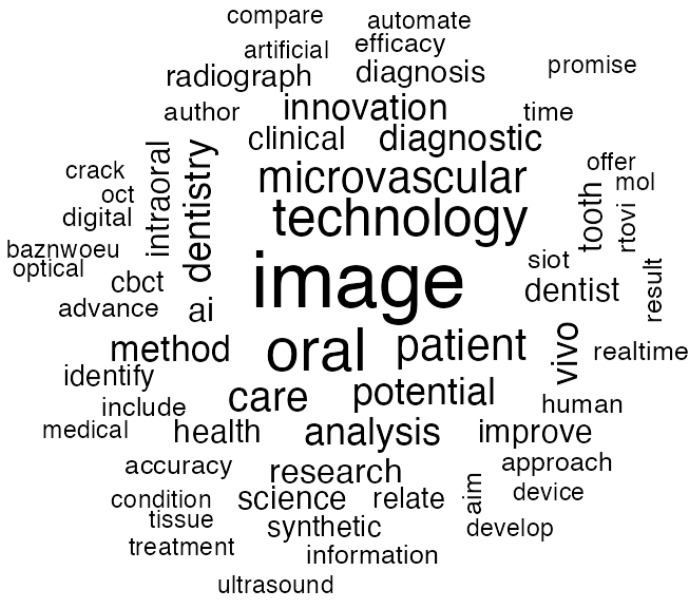
Word cloud visualization of frequently and repeatedly used words about biomaterials.

Biomaterials have emerged as a pivotal influence in contemporary dentistry, significantly altering the methodologies employed by practitioners in the planning and implementation of treatments. These materials provide effective responses to clinical dilemmas while also facilitating avenues for innovation, thereby enhancing both functional results and patient satisfaction. Figure 8 presents the most common terms found in the abstracts of imaging-related articles. Topics appearing in darker and larger fonts indicate higher frequency.

The application of “image technologies” marks a big step in the development of biomaterials. Amezua et al. [28] have shown how facial scanning methods can change the production of prosthetic devices. On acquiring complex anatomical data, these technologies make it possible to produce tailor-made biomaterials that would easily fit the individual’s specific anatomical features. This precision enhances both the aesthetic and functional reliability of the devices, making treatments truly personalized and effective.

In the meantime, “innovative” imaging technologies, such as CBCT, have shown significant importance in the use of biomaterials in “oral care”. Shujaat et al. [29] stated that models generated from CBCT help in aligning biomaterial-based restorations with the high standards required in contemporary dentistry. These tools are particularly relevant in minimally invasive procedures, where accuracy is crucial for the preservation of natural tissues and the achievement of optimal results.

Digital workflows have significantly improved the adaptability and effectiveness of biomaterial applications. Xepapadeas et al. [30] examined cutting-edge techniques that incorporate biomaterials into “dentistry” treatments with unparalleled accuracy and efficiency. These techniques involve the utilization of high-resolution intraoral scanners for obtaining digital impressions, 3D modeling software for the development of customized orthodontic appliances, and 3D printers for the direct production of components. These workflows not only improve production efficiency but also ensure better adherence to clinical standards while significantly reducing treatment times, thus creating a more efficient and predictable experience for patients.

The use of magnification modalities has added a new level of precision to the use of biomaterials. Rashkova et al. [31] assessed how dental operating microscopes are advancing the use of modern biomaterials. Magnification allows for deliberate material placement and manipulation, minimizing errors and maximizing the longevity and success rate of restorations.

These are not mere technological strides but also reflect a deeper commitment to raising the bar in the delivery of dental care. The integration of biomaterials with state-of-the-art imaging and digital tools shows how dentistry is moving toward more patient-centered and scientifically grounded practices. Each innovation builds on a foundation of rigorous research and clinical expertise, ensuring that new solutions effectively address real-world challenges.

As investigations progress to expand the potential capabilities of biomaterials, the opportunities for their utilization in the field of dentistry are broadening. This continuous development underscores the significance of collaborative efforts across various disciplines and the necessity of ongoing education, thereby ensuring that the dental profession remains a leader in medical advancements while adhering to the fundamental principles of high-quality care and patient welfare.

## 4. Discussion

### 4.1. Impact of Emerging Imaging Technologies on Dental Practice

The evolution of diagnostic methods and imaging technologies has significantly transformed dentistry, enhancing diagnostic accuracy, efficiency, and personalized treatment planning. While traditional techniques have reliably served the field, the introduction of advanced imaging systems has expanded the ability to analyze complex dental structures and offer minimally invasive treatments. CBCT, for instance, has redefined implantology and surgical planning by providing high-resolution three-dimensional imagery, allowing for the precise visualization of dental and maxillofacial structures [5]. Similarly, AI-driven analysis of dental images has demonstrated the potential to automate diagnostics, reducing human error and improving efficiency.

However, these advancements come with notable challenges. As highlighted by Bhat et al. [32], integrating 3D printing with CBCT imaging enhances personalized treatment planning but requires substantial financial investments and professional training. This reality raises concerns about the accessibility of these innovations, particularly in regions with limited economic resources. A balanced approach, leveraging conventional methods for preliminary diagnostics while reserving advanced tools for complex cases, may help optimize resource allocation and ensure broader access to high-quality dental care.

### 4.2. Challenges and Limitations of Adoption

Despite their transformative potential, emerging technologies face several adoption barriers. High costs and maintenance expenses remain major deterrents, especially for smaller dental practices. Additionally, mastering these tools necessitates specialized training, creating a steep learning curve for practitioners. Beyond financial and educational concerns, over-reliance on AI-driven diagnostics presents another challenge. While AI algorithms can enhance efficiency, they are not infallible; issues such as false positives and misdiagnoses require ongoing validation through clinical trials and human oversight.

Ethical concerns are also increasingly relevant. Data privacy, biases in AI algorithms, and regulatory uncertainties need urgent attention. For example, AI systems trained on datasets lacking diversity may produce biased diagnostic outputs, impacting clinical decision-making. Furthermore, there is a growing need for global frameworks to standardize the integration of AI and imaging technologies in dental practice, ensuring ethical deployment and equitable patient care.

### 4.3. Future Directions for Research and Policy Considerations

While this review highlights the potential of advanced imaging technologies, it also identifies key research gaps that must be addressed. Future studies should prioritize conducting large-scale clinical trials to assess the long-term efficacy and safety of AI-assisted diagnostics. Additionally, comparative cost-effectiveness analyses are needed to determine the financial viability of implementing CBCT, AI, and laser fluorescence in routine practice. Another essential area of focus is the development of strategies to integrate new technologies into standardized clinical workflows without disrupting existing practices. Finally, regulatory guidelines must be established for the ethical use of AI, with particular emphasis on bias mitigation and data security.

Addressing these research gaps will be crucial to ensuring that the benefits of technological advancements in dentistry are both sustainable and inclusive. By fostering interdisciplinary collaboration and prioritizing patient-centered outcomes, the dental field can continue evolving while maintaining ethical and economic considerations at the forefront. The integration of AI, CBCT, and other emerging imaging technologies marks a new era in dental diagnostics, characterized by unparalleled precision and individualized treatment approaches. However, overcoming barriers related to cost, accessibility, and ethical considerations remains essential to their widespread adoption. Ensuring that these innovations are implemented responsibly, with adequate training and regulatory oversight, will be fundamental to maximizing their impact. By addressing these challenges through targeted research and policy development, the future of dental diagnostics can be both technologically advanced and widely accessible.

## 5. Conclusions

The field of dental medicine is undergoing a profound transformation driven by advancements in diagnostic methods and imaging technologies. Innovations such as CBCT, artificial intelligence, and biomarker-based diagnostics have significantly improved diagnostic accuracy and facilitated personalized, minimally invasive treatment approaches. However, despite these benefits, several challenges persist, including high implementation costs, the need for specialized training, and disparities in global access to these technologies. Addressing these barriers is crucial to ensuring the equitable integration of these innovations into clinical practice.

Beyond their technical advantages, these technologies play a pivotal role in improving early disease detection, optimizing treatment plans, and enhancing patient outcomes. Nevertheless, concerns surrounding data privacy, bias in AI algorithms, and regulatory oversight require urgent attention. Without robust ethical frameworks, the risk of diagnostic errors, biased AI-driven decisions, and patient data vulnerabilities remains a significant concern. Future research should prioritize the development of standardized protocols for AI-based diagnostics, large-scale clinical trials to validate emerging imaging technologies, and cost-effectiveness analyses to evaluate their long-term viability in diverse healthcare settings.

This review also highlights key limitations of the existing literature. Many studies suffer from small sample sizes, methodological inconsistencies, and limited longitudinal data. These factors restrict the generalizability of findings and emphasize the need for more rigorous, large-scale investigations. Additionally, the lack of comparative studies on cost-effectiveness hinders a comprehensive assessment of the feasibility of implementing these technologies in everyday dental practice.

Moving forward, multidisciplinary collaboration will be essential to refine these technologies and integrate them seamlessly into clinical workflows. By developing practical, scalable solutions and addressing economic and ethical barriers, dental medicine can fully harness the potential of emerging diagnostic tools. Ultimately, this progress will contribute to enhanced patient care, improved professional training, and a more equitable distribution of advanced dental technologies worldwide.

## Data Availability

Datasets are available on request: The raw data supporting the conclusions of this article will be made available by the authors, without undue reservation.
